# Conducting Rubber Anisotropy of Electrophysical and Mechanical Properties

**DOI:** 10.3390/polym17040492

**Published:** 2025-02-14

**Authors:** Stanislav Makhno, Xianpeng Wan, Oksana Lisova, Petro Gorbyk, Dongxing Wang, Hao Tang, Yuli Shi, Mykola Kartel, Kateryna Ivanenko, Sergii Hozhdzinskyi, Galyna Zaitseva, Maksym Stetsenko, Yurii Sementsov

**Affiliations:** 1 Ningbo Sino-Ukrainian New Materials Industrial Technologies Institute, Kechuang Building, N777 Zhongguan Road, Ningbo 315211, China; stmax@ukr.net (S.M.); wxp522082973@163.com (X.W.); wangdongxing518@163.com (D.W.); tanghao@nbut.edu.cn (H.T.);; 2 O. Chuiko Institute of Surface Chemistry NAS of Ukraine, 17, General Naumov Street, 03164 Kyiv, Ukrainegorbuk@isc.gov.ua (P.G.); nikar@kartel.kiev.ua (M.K.); 3 School of Materials and Chemical Engineering, School of Safety Engineering, Ningbo University of Technology, 201 Fenghua Road, Ningbo 315211, China; 4 Institute of Macromolecular Chemistry, NAS of Ukraine, 48 Kharkiv Highway, 02160 Kyiv, Ukraine; 5 Department of Analytical, Physical and Colloid Chemistry, Pharmaceutical Faculty, Bogomolets National Medical University, 13 T. Shevchenko blvd., 01601 Kyiv, Ukraine; farmanalit@ukr.net (S.H.); analyt.chemistry@nmu.ua (G.Z.); 6 School of Science, Westlake University, Hangzhou 310024, China; stetsenkomax@gmail.com; 7 Institute of Natural Sciences, Westlake University, Hangzhou 310024, China

**Keywords:** conductive rubber, multiwall carbon nanotubes, electrical conductivity, dielectric permittivity, tensile strength, percolation system

## Abstract

The aim of this work was to determine the anisotropy of the electrophysical and mechanical properties of rubber reinforced with a hybrid filler CNTs&CB (carbon nanotubes and carbon black) as a function of CNT content and the technological parameters of the production process. A significant difference in electrical conductivity (σ) and dielectric permittivity (ε) in three perpendicular directions was found for CNT concentrations ranging from 0 to 0.007 in volume fraction. The highest values of σ and ε were observed in the calendering direction, with slightly lower values in the perpendicular direction. This effect was attributed to the orientation of polymer molecules and CNTs along the direction of movement during calendering, as well as the disruption of the cluster structure in the transverse direction. Although the calculated percolation threshold values of the investigated system differed slightly, a correlation was observed between the mechanical and electrophysical properties of CNTs&CB rubber. This correlation enables rubber products to be designed with optimal properties tailored to the desired direction.

## 1. Introduction

In the contemporary world, electromagnetic radiation across a broad spectrum of frequencies, ranging from very low to extremely high power levels, is ubiquitous. Therefore, products made from electrically conductive polymer composites, including rubber, are widely used as electromagnetic shields, electrostatic coatings, sensors, etc. [1,2,3,4,5,6]. Special grades of carbon black, graphite, carbon fibers, and powders of nickel, copper, silver, and other metals serve as conductive fillers for such systems. However, carbon-based fillers of various morphologies and origins have gained widespread application due to their cost-effectiveness, ease of processing, corrosion resistance, and high reproducibility of technical results [7,8,9]. Recently, the potential of nano-sized fillers, such as nanoclay, nanosilica, carbon nanotubes, and graphene nanoplatelets, as well as their combinations with macro- and micro-fillers (hybrid fillers) for reinforcing polymer matrices [8,9,10,11,12,13,14,15,16,17,18,19,20,21,22], including rubber [5,6,8,9,23,24,25,26,27,28,29,30,31,32,33,34,35,36], has been extensively studied. In our previous study [31], we conducted a comprehensive analysis of the aforementioned studies and demonstrated that the reinforcement of rubbers with a hybrid filler of multiwall carbon nanotubes and carbon black (CNTs-CB) significantly enhances the mechanical characteristics of rubbers. Today, conductive rubber products play a crucial role in applications such as electromagnetic shields and electrostatic coatings. It is known that CNTs exhibit high electrical conductivity (σ), mechanical strength (P), and a large aspect ratio, making them promising as conductive fillers. The electrical conductivity of CNT-filled rubber depends on the formation of secondary (chain-like) structures, which are influenced by the CNT content of CNT and various technological factors.

It should be noted that rubber compounds have been in development for almost two hundred years. Initially, they were based on natural rubbers and, later, synthetic rubbers were introduced. Rubber compounds are complex, multi-component systems that can contain up to 12–18 components, including various additives such as stabilizers, activators, antioxidants, and modifiers. The introduction of carbon black as a filler transformed rubber into an elastomeric system with exceptional elastic properties. Extensive research spanning multiple generations has led to the optimization of rubber formulations for various applications through systematic investigations [9,22,23,24,25,26,27,28,29,30,37,38,39,40,41]. Therefore, for our study, we selected a well-known composition based on the commercially produced acrylonitrile-butadiene rubber copolymer NBR 3365 [31]. This system was analyzed from the perspective of polystructural theory [42,43]. The essence of this approach lies in identifying multiple interdependent structures within a single system, spanning from the atomic (nanoscale) level to coarser component structures (macroscale) that interconnect in a hierarchical manner (‘structure within structure’ or ‘composite within composite’), as was previously analyzed in [31]. Another key assumption was that, in a dense material, the rubber should completely wet the system and form a layer of a certain thickness on the filler surface, approximated as twice the diameter of the CNT [31].

The aim of this work was to determine the anisotropy of the electrophysical and mechanical properties of rubber reinforced with a hybrid CNTs&CB filler as a function of the CNT content near the calculated optimal value and the technological parameters of the production process.

## 2. Materials and Methods

**Figure 1:**
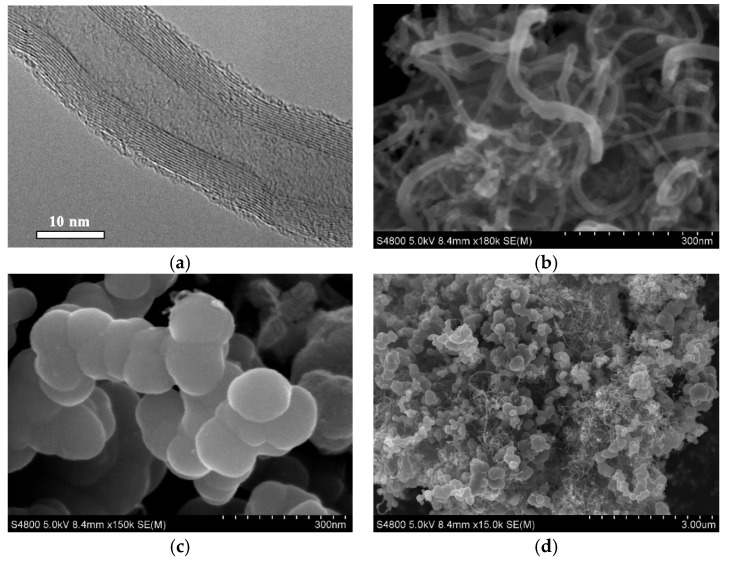

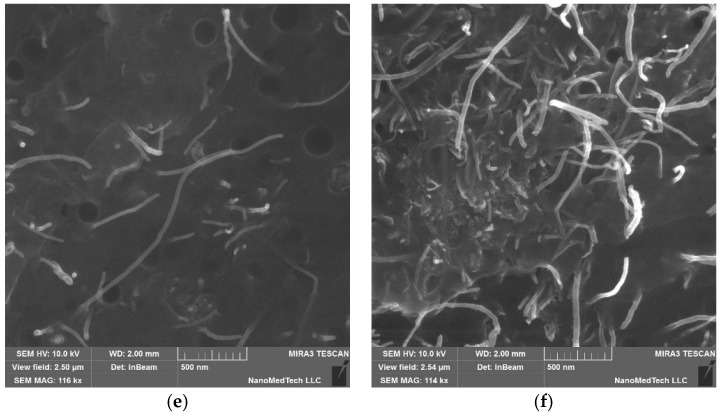
(**a**) TEM image of CNTs; SEM images: (**b**) agglomerations of CNTs, (**c**) carbon black, (**d**) composite CB+CNTs, and (**e**,**f**) cross-sectional view of the rubber compound.

The acrylonitrile-butadiene rubber copolymer NBR 3365 with carbon black N550 was used as the initial material [44] (Figure 1 and Table 1). Multiwall carbon nanotubes (Figure 1a,b) (TU U 24.1-03291669-009:2009 [45] were synthesized via chemical vapor deposition (CVD) using propylene as the carbon source in a rotating reactor. The characteristics of the obtained CNTs were as follows: average diameter—from 10 to 20 nm; specific surface area (determined by Ar adsorption)—from 200 to 400 m^2^/g; bulk density—from 20 to 40 g/dm^3^ [45,46]. Prior to incorporation into the rubber matrix, CNTs and carbon black were first dispersed in ethanol. The prepared suspensions were then mixed at a ratio calculated according to Equations (1)–(3) [31]:

(1) $$m_{cb}=m_{cb}^{0}-\frac{S_{cb}}{S_{CNT}}\times m_{CNT},$$

(2) $$m_{CNT}=\frac{\frac{m_{rub}}{2\rho_{rub}\cdot d_{CNT}\cdot S_{CNT}}-m_{cb}^{0}\cdot \frac{S_{cb}}{S_{CNT}}}{1-{\left(\frac{S_{cb}}{S_{CNT}}\right)}^{2}}$$

(3) $$m_{cb+CNT}=m_{cb}^{0}-\frac{S_{cb}}{S_{CNT}}\times m_{CNT}+m_{CNT}$$

where *m_cb_* is the carbon black content in parts per hundred rubber; *S_cb_* is the carbon black specific surface area; *S_CNT_* is the specific surface area of CNT; *m_CNT_* is the CNT content in parts per hundred rubber; *m*^0^*_cb_* is the carbon black content in parts per hundred rubber in the original optimized composition of the rubber; and *m_rub_* and *ρ_rub_* are the mass and density of rubber, respectively.

The suspensions were then further sonicated using an Ultrasonic Disperser-M900T, dried to a constant weight, and dispersed in the mixer (Figure 1d). According to [47], such pre-mixing of fillers can enhance the dispersion within elastomer compositions, ultimately leading to improved material characteristics.

The composition of the initial rubber system is presented in Table 1. The technological process of rubber production is described in detail in [31] and was carried out using equipment from the TKW Rubber Taishun Seal factory (Ningbo, China) [44]. For the calculation of the expected optimal CNT content, NBR 3365 rubber with a density of 0.915 g/cm^3^ was used. The CNTs had a specific surface area of 230 m^2^/g and a diameter of 15 nm. The specific surface area of N550 carbon black was considered at values of 40.0, 42.0, and 44.0 m^2^/g.

For a carbon black content of 80 phr (parts per hundred rubber), the calculated CNT content range was 3.6, 2.4, 1.2, 0.6, 0.4, 0.3, 0.2, and 0.1 phr. In terms of the entire rubber compound, this corresponded to approximately half these values per 100 parts of the total system. In this study, eight series of samples with different CNT contents were analyzed (Table 2).

The curing characteristics were measured experimentally using a GB/T16584 (MN-4010B) (GOTECH Testing Machines Co., Ltd., Dongguan, China) [44].

The curing parameters of the obtained samples were as follows:

*t_s_*_1_ (min)—scorch time;

*t_c_*_90_ (min)—optimum cure time;

*M_L_* (dN·m)—minimum torque;

*M_H_* (dN·m)—maximum torque;

*R_v_* (min^−1^)—cure rate index, defined as

$$R_{v}=\frac{100}{\left(t_{c90}-t_{s1}\right)}.$$

The density of the samples was measured using a Density Tester AKD-310A (Yangzhou Aikeide Instrument Co., Ltd., Yangzhou, China), with a measurement error of 2%.

The hardness of the samples was determined using Shore hardness testing, in accordance with ISO 7619-1:2010, and the measurements were conducted at a certified testing facility in China [44].

The structure of multiwall CNTs was analyzed using a JEM-2100F (JEOL, Ltd., Tokyo, Japan) 200 kV FE (field emission) analytical electron microscope with 1 Å resolution (Figure 1a). The morphology and distribution of CNTs, carbon black, and carbon black–CNT systems (Figure 1b–d) were analyzed on a Hitachi S-4800 high-resolution scanning electron microscope (Hitachi High-Technologies Corporation, Tokyo, Japan; resolution of 2.0 nm at 1 kV for low-voltage applications). The cross-sectional morphology of the rubber compound (Figure 1e,f) was studied using a Tescan Mira 3 electron microscope (Tescan, Brno, Czech Republic) equipped with a high-resolution Schottky field-emission emitter (resolution 1.2 nm at 30 kV).

To investigate the electrophysical and mechanical characteristics, samples of varying geometries and sizes were fabricated.

The dielectric properties in the microwave range were measured on samples with dimensions of 23 mm × 10 mm × 5 mm.

For low-frequency measurements, samples ranging in size from 5 mm × 5 mm × 5 mm to 20 mm × 20 mm × 5 mm were used to ensure the appropriate sensitivity of the RLC Meter 880 BK PRECISION, which operates at five frequencies: 0.1, 0.12, 1, 10, and 100 kHz.

The real (ε′) and imaginary (ε″) components of the complex dielectric permittivity were measured at 10 GHz using the interferometer (RFK 2–18, for measuring the phase differences) and the standing wave meter (R2-60) via an electrodeless method on samples of 23 mm × 10 mm × 5 mm [14].

Graphite electrodes were used for electrical conductivity measurements with an experimental error not exceeding 5%.

Samples for mechanical tests were prepared in the dumbbell shape according to ISO 37:2017 and the measurements were conducted at a certified testing facility in China [44]. The tensile test was conducted at room temperature (26 °C) using an electro-universal testing machine AI-7000-MT (GOTECH Testing Machines Co., Ltd., Dongguan, China).

**Figure 2:**
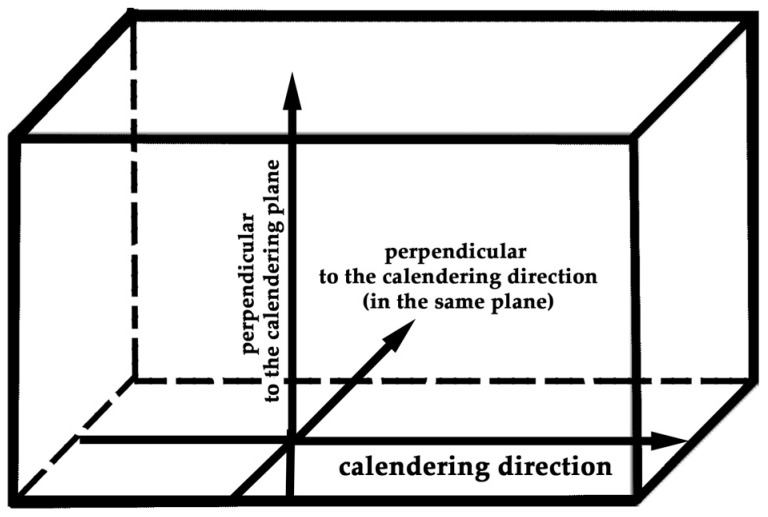
Scheme for measuring electrophysical and mechanical characteristics.

Figure 2 illustrates the measurement directions for both electrophysical and mechanical properties.

## 3. Results and Discussion

### 3.1. SEM Analysis

The morphology of the synthesized pure CNTs was visualized using TEM and SEM microscopes, as shown in Figure 1a and Figure 1b, respectively. The nanotubes observed are homogeneous, with a diameter of approximately 15 nm, which is clearly visible in the TEM image (Figure 1a). Importantly, the image in Figure 1a shows tubes with smooth surfaces and no apparent defects. Figure 1b demonstrates that the MWCNTs exist as aggregates with distinct long tubular structures. As observed in Figure 1c, carbon black appears as typical assembled spherical particles, with a size of less than 150 nm.

The SEM images of the nanocomposites consisting of carbon black and carbon nanotubes show the decoration of the carbon black surface (Figure 1d).

Furthermore, the SEM images of the cross-sectional view of the rubber compound (Figure 1e,f) show a homogeneous distribution of CNTs in the rubber, with no significant morphological differences between the synthesized Sample N5 and Sample N8, other than the CNT concentrations (0.6 and 3.6, respectively, as shown in Table 2).

### 3.2. Curing Process

The influence of filler content on the vulcanization process of rubber compounds was evaluated by analyzing the vulcanization characteristics, including scorch time (*t_s_*_1_), optimum cure time (*t_c_*_90_), cure rate index (*R_v_*), minimum torque (*M_L_*), and maximum torque (*M_H_*), as presented in Table 2.

As demonstrated in Table 2, samples with higher CNT concentrations exhibit a prolonged scorch time, vulcanization (cure) time, and both maximum and minimum torques. Consequently, the cure rate index for these samples is lower.

The shortest scorch time and optimum cure time were observed in the initial samples and those with lower CNT content, which also exhibited a higher cure rate index (Table 2). This indicates that the curing process of materials with a lower CNT content is faster, meaning that a small addition of CNTs can accelerate the vulcanization process. The observed acceleration can be attributed to the enhanced thermal conductivity of the CNT-reinforced rubber matrix, which improves heat transfer throughout the composite, leading to faster cross-linking reactions. As shown in Table 2, the addition of CNTs resulted in an increase in both minimum and maximum torque. The minimum torque is associated with the viscosity of the rubber compounds prior to vulcanization, clearly indicating higher CNT loading compared to the rubber matrix, a trend also observed in previous studies [6,28,48]. The maximum torque corresponds to the viscosity of the cured rubber compound, which is directly related to the cross-link density. Both CB and CNTs exhibit high compatibility with the rubber matrix, forming strong interfacial adhesion between the matrix and the carbon fillers. According to [6], the thin rubber layer at the rubber–filler interface is strongly bound to the fillers through physical adsorption or chemisorption, behaving like a glassy-state polymer. This phenomenon significantly enhances both the strength and density of cross-links [6,28,48].

### 3.3. Electrical Properties

**Figure 3:**
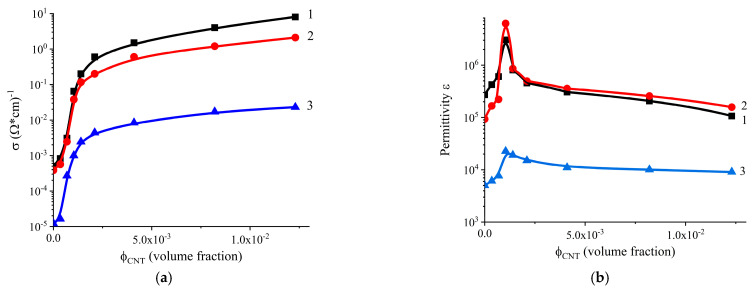
Dependence of electrical conductivity (**a**) and dielectric permittivity (**b**) at 100 Hz on the content of CNTs in three perpendicular directions of measurement: Curve 1—in the direction of calendering; Curve 2—perpendicularly, in the same plane; Curve 3—perpendicular to the calendering plane.

The results of the electrical conductivity (σ) and the real component of the complex dielectric permittivity (ε′) measurements are presented in Figure 3. As observed, both parameters exhibit a nonlinear dependence on CNT content, which can be attributed to the percolation transition phenomenon.

The analysis of the obtained results from the perspective of percolation theory according to Equations (4) and (5) [49,50] was carried out:

(4) $$\sigma =\sigma_{i}{\left(\phi -\phi_{c}\right)}^{t},\;\;\;at\;\phi >\phi_{c},$$

(5) $$\varepsilon^{\prime }={\varepsilon^{\prime }}_{\;i}{\left(\phi_{c}-\phi \right)}^{-v},\;\;at\;0<\phi <\phi_{c},$$

where *σ_i_* and *ε′_i_* are the electrical conductivity and dielectric permittivity of the filler; ϕ is the volume content; *ϕ_c_* is the content value corresponding to the percolation threshold; *t* and *v* are power factors; and *t* is the critical conductivity index, which mainly depends on the topological dimension of the system and does not depend on the structure of the particles forming the clusters and their interaction (for a three-dimensional system, the theoretical value of t is in the range from 1.6 to 2.06 [50,51,52]).

The percolation threshold is approximately 0.0009 in volume fraction for all directions of electrical conductivity measurement. As MWCNTs are incorporated into the rubber matrix, an increase in electrical conductivity is observed across the entire range of MWCNT concentrations studied [36,48,53,54]. Fluctuations within the margin of experimental error may occur before reaching the percolation threshold. Below this threshold, electrical conductivity increases with frequency due to the hopping conduction mechanism [55,56]. The frequency dependence of the real component of the specific electrical conductivity is described by the following relation:

(6) $$\sigma \left(\omega \right)=\sigma_{dc}+A\omega^{n},$$

where *A* and *n* are parameters that depend on temperature and composite composition. In similar composite systems, the value of *n* is 0.8.

For MWCNT concentrations above the percolation threshold, electrical conductivity becomes independent of frequency.

**Figure 4:**
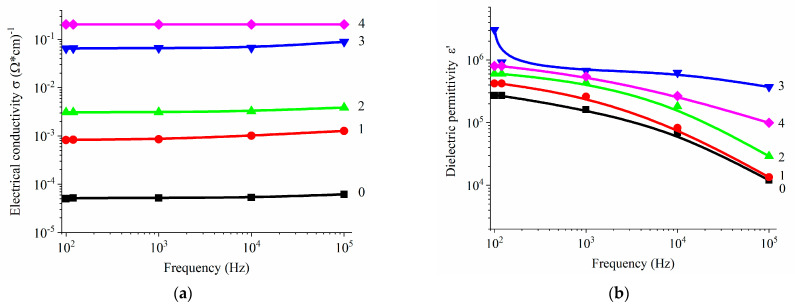

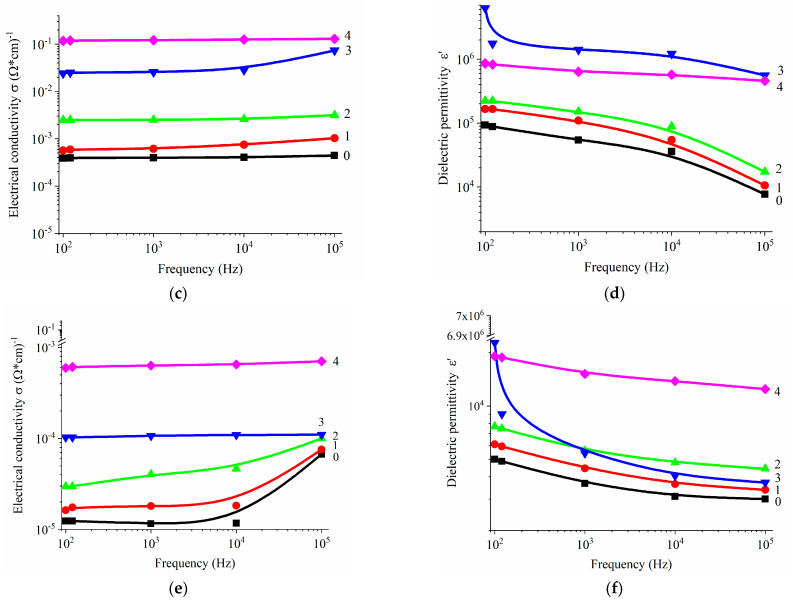
Dependence of electrical conductivity (**a**,**c**,**e**) and dielectric permittivity (**b**,**d**,**f**) on frequency in three perpendicular directions of measurement: (**a**,**b**) in the direction of calendering; (**c**,**d**) perpendicularly, in the same plane; (**e**,**f**) perpendicular to the calendering plane. For the content of CNT: 0 (Curve 0), 0,00035 (Curve 1), 0,00069 (Curve 2), 0,00104 (Curve 3), 0,0014 (Curve 4) v.f. to the entire composite.

A significant difference in electrical conductivity and dielectric permittivity is observed in three perpendicular directions across the range of CNT concentrations from 0 to 0.007 in volume fraction. The highest values of both electrical conductivity and dielectric permittivity are observed in the calendering directions, while significantly lower values are seen in the perpendicular direction (Figure 4).

**Figure 5:**
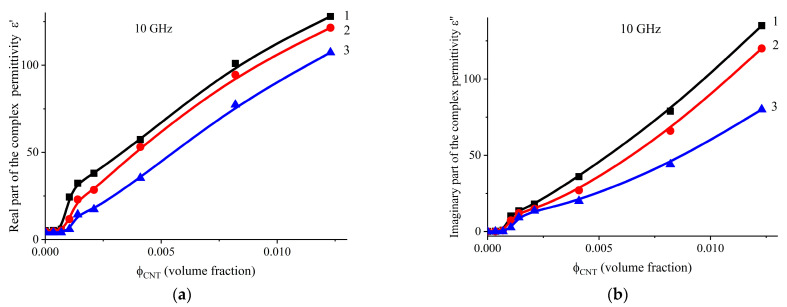
Dependence of the real (**a**) and imaginary (**b**) components of the complex dielectric permittivity on the content of CNTs in three perpendicular directions of measurement: Curve 1—in the direction of calendering; Curve 2—perpendicularly, in the same plane; Curve 3—perpendicular to the calendering plane.

The components of the complex dielectric permittivity exhibit high values, indicating a significant level of interaction between the system components and a uniform distribution of the conductive component within the composite. This behavior is associated with the orientation of polymer and filler molecules, including CNTs [48], in the direction of movement, as well as the disruption of the cluster structure in the transverse direction. Although the calculated percolation threshold values for the considered system differ only slightly, the maxima of the dielectric permittivity observed in the concentration-dependent curves can be attributed to the formation of the largest interfacial area between the CNTs and the polymer binder. A noteworthy result is the large difference in the real and imaginary components of the dielectric permittivity, depending on the direction of measurement, as shown in Figure 5.

### 3.4. Physical-Mechanics Characteristics

Improving the physical and mechanical properties of rubber composites has always been an important task for researchers. As part of this work, tensile strength, density, and hardness were measured.

**Figure 6:**
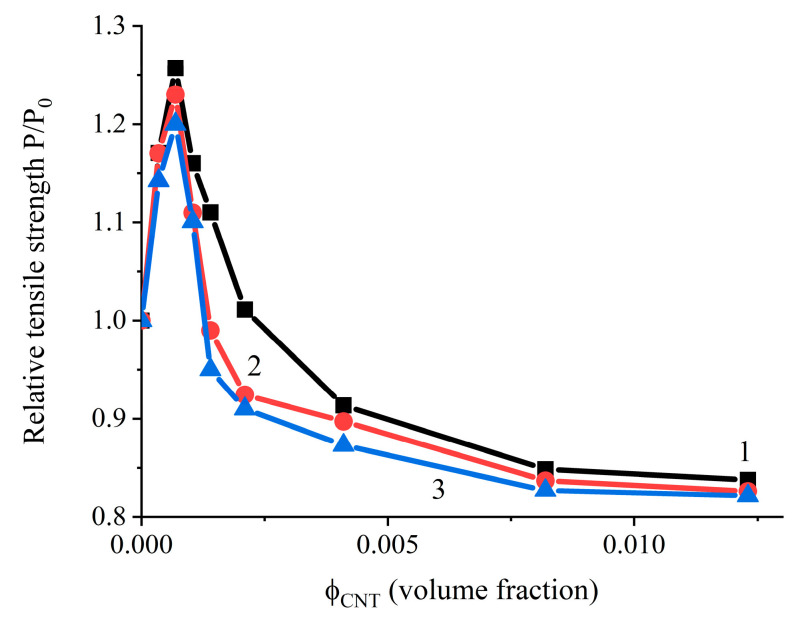
Dependence of the relative tensile strength on the content of CNTs in three perpendicular measurement directions: Curve 1—in the direction of calendering; Curve 2—perpendicularly, in the same plane; Curve 3—perpendicular to the calendering plane.

Figure 6 shows the ratio of the tensile strength limit to the strength limit of the sample without the filler, relative to the CNT content measured in different directions. To measure the strength limit perpendicular to the calendering plane, a sample approximately 8 mm thick was prepared.

Maximum values are observed in all three directions. In these cases, the volume fraction of CNT concentrations is approximately 0.0007, which also correlates with the percolation threshold data (0.0009). Additionally, these experimental values are in agreement with the expected CNT content (0.3 phr, Table 2) for maximum reinforcement efficiency calculated according to the proposed model. Analyzing the results shown in Figure 3 and Figure 6, it can be concluded that there is a relationship between the CNT content at the maximum tensile strength and percolation threshold. By lowering the percolation threshold in polymer systems filled with CNTs, it is possible to achieve high levels of electrical conductivity and dielectric losses in the ultra-high-frequency range of electromagnetic radiation with a lower CNT content, while simultaneously attaining higher strength parameters [14].

It should also be noted that the addition of a small amount of CNTs to rubber slightly increases its density r and has minimal impact on the hardness of the compound (Table 3).

The primary objective was to compare the properties of mass-produced rubber with rubber to which a small amount of MWCNTs was added. It was found that the mechanical characteristics of the rubber improve within a narrow range of MWCNT concentrations. The concentration of MWCNTs at which the mechanical properties begin to decrease is approximately 0.001, which is consistent with the literature data [14,31,57,58,59,60,61,62,63].

## 4. Conclusions

Elastomer compositions based on NBR 3365 rubber with technical carbon black N550 of a standard formulation, combined with a specially calculated amount of CNTs, were obtained. It was confirmed that the relationship between the maximum electrical and mechanical properties occurs within a narrow range of CNT concentrations.

Anisotropic features are evident in the electrical properties of the elastomeric composites, with a noticeable difference between the properties in the calendering direction and those in the perpendicular directions. Specifically, electrical conductivity (σ) and dielectric permittivity (ε) exhibit significantly higher values in the calendering direction due to the orientation of the polymer and CNT molecules during processing. This directional dependence emphasizes the importance of the processing technique in determining the final properties of the composite material.

However, no such anisotropy is observed in the mechanical properties of the composites in different directions, suggesting that the incorporation of CNTs does not significantly affect the structural integrity of the elastomer compositions. Mechanical performance remains stable despite the directional dependence of electrical characteristics. The calculated percolation thresholds for the CNTs within the composite show minimal variation across directions, allowing for the prediction of the impact of CNT addition on conductivity and dielectric properties with high accuracy. By adjusting the CNT concentration and processing conditions, it is possible to optimize the balance between the electrical and mechanical performance of the rubber, making it suitable for various applications where both properties are critical.

Furthermore, understanding the anisotropic behavior of the composites can be leveraged to design products that meet specific requirements for conductivity and mechanical strength, making these materials highly versatile and valuable in a wide range of applications.

## Figures and Tables

**Table 1:** Composition of the rubber compound.

Material Title	NBR 3365	Zink Oxide	Stearic Acid 1801	Antioxidant 4010NA	Releasing Agent 935P	Carbon Black N550	Plasticizer			
							DOP	S	CZ	TMTD
Content, phr	100	5	1.5	2	2	80	10	0.5	1.5	2

**Table 2:** Correlation of CNT content and curing characteristics: scorch time *t*_10_, optimum cure time *t_c_*_90_, minimum torque *M_L_*, maximum torque *M_H_*, and curing rate index *R_v_*.

Sample	Content CNTs, phr	ϕ_CNT_, Volume Fraction	*t*_10_, s	*t*_*c*90_, s	*M_L_*, dN·m	*M_H_*, dN·m	*R_v_*, s^−1^
0	0	0	32	68	2.44	18.19	2.78
1	0.1	0.00035	32	68	2.53	19.78	2.78
2	0.2	0.00069	30	60	2.68	19.82	3.33
3	0.3	0.00104	31	62	2.47	19.18	3.22
4	0.4	0.0014	32	61	2.55	20.03	3.45
5	0.6	0.0021	42	106	2.93	22.85	1.56
6	1.2	0.0041	45	104	3.1	24.86	1.7
7	2.4	0.0082	43	101	3.14	23.47	1.72
8	3.6	0.0123	43	100	3.8	24.9	1.75

**Table 3:** Density and hardness (Shore A) for rubber compounds.

**Content CNTs ϕ_CNT_, v.f.**	0	0.00035	0.00069	0.00104	0.0014	0.0021	0.0041	0.0082	0.0123
**Density ρ, g/cm^3^**	1.250	1.251	1.252	1.259	1.260	1.265	1.270	1.270	1.265
**Hardness, Shor A**	77	77	77	78	78	76	77	78	80

## Data Availability

Data are available in this article.

## Abbreviations

The following abbreviations are used in this manuscript:

NBR	Acrylonitrile-butadiene rubber
CB	Carbon black
CNTs	Carbon nanotubes
v.f.	Volume fraction
MWCNTs	Multiwall carbon nanotubes
